# Improving furfural tolerance in a xylose-fermenting yeast *Spathaspora passalidarum* CMUWF1–2 via adaptive laboratory evolution

**DOI:** 10.1186/s12934-024-02352-x

**Published:** 2024-03-13

**Authors:** Thanyalak Saengphing, Pachara Sattayawat, Thitisuda Kalawil, Nakarin Suwannarach, Jaturong Kumla, Mamoru Yamada, Watanalai Panbangred, Nadchanok Rodrussamee

**Affiliations:** 1 Department of Biology, Faculty of Science, Chiang Mai University, Chiang Mai, 50200 Thailand; 2 Center of Excellence in Microbial Diversity and Sustainable Utilization, Chiang Mai University, Chiang Mai, 50200 Thailand; 3 Department of Biological Chemistry, Faculty of Agriculture, Yamaguchi University, Yamaguchi, 753-8515 Japan; 4 Life Science, Graduate School of Science and Technology for Innovation, Yamaguchi University, Ube, 755-8611 Japan; 5 Research Center for Thermotolerant Microbial Resources, Yamaguchi University, Yamaguchi, 753-8515 Japan; 6 King Mongkut’s University of Technology Thonburi, Bangkok, 10140 Thailand

**Keywords:** Adaptive laboratory evolution, Evolutionary engineering, Furfural tolerance, Lignocellulosic biomass, *Spathaspora passalidarum*

## Abstract

**Background:**

*Spathaspora passalidarum* is a yeast with the highly effective capability of fermenting several monosaccharides in lignocellulosic hydrolysates, especially xylose. However, this yeast was shown to be sensitive to furfural released during pretreatment and hydrolysis processes of lignocellulose biomass. We aimed to improve furfural tolerance in a previously isolated *S. passalidarum* CMUWF1−2, which presented thermotolerance and no detectable glucose repression, via adaptive laboratory evolution (ALE).

**Results:**

An adapted strain, AF2.5, was obtained from 17 sequential transfers of CMUWF1−2 in YPD broth with gradually increasing furfural concentration. Strain AF2.5 could tolerate higher concentrations of furfural, ethanol and 5-hydroxymethyl furfuraldehyde (HMF) compared with CMUWF1−2 while maintaining the ability to utilize glucose and other sugars simultaneously. Notably, the lag phase of AF2.5 was 2 times shorter than that of CMUWF1−2 in the presence of 2.0 g/l furfural, which allowed the highest ethanol titers to be reached in a shorter period. To investigate more in-depth effects of furfural, intracellular reactive oxygen species (ROS) accumulation was observed and, in the presence of 2.0 g/l furfural, AF2.5 exhibited 3.41 times less ROS accumulation than CMUWF1−2 consistent with the result from nuclear chromatins diffusion, which the cells number of AF2.5 with diffuse chromatins was also 1.41 and 1.24 times less than CMUWF1−2 at 24 and 36 h, respectively.

**Conclusions:**

An enhanced furfural tolerant strain of *S. passalidarum* was achieved via ALE techniques, which shows faster and higher ethanol productivity than that of the wild type. Not only furfural tolerance but also ethanol and HMF tolerances were improved.

**Supplementary Information:**

The online version contains supplementary material available at 10.1186/s12934-024-02352-x.

## Background

Concerns over the shortage of petroleum and its negative impacts on the environment have prompted the world to search for alternative chemical and fuel supply sources. Microbial cell factories have been one of the most studied alternatives by which microorganisms are used as a factory to produce attractive chemicals and fuels via their native metabolism or synthetic design [1, 2]. In the case of ethanol as biofuel widely used in many applications [3].

Yeasts have long been used as the production host with their native ability to produce ethanol [4]. *Spathaspora passalidarum* is a yeast well-known for its ability to efficiently convert xylose to ethanol [5–7]. This trait is considered highly important for ethanol production from agricultural residues, as xylose is the second most abundant sugar in such materials [8]. High-value chemical production from these feedstocks is favorable and considerably sustainable as it does not compete with food sources and land uses. Agricultural residues such as rice straw, wheat straw, and bagasse consist mainly of lignocellulosic biomass arranged in a complex structure, which roughly contains 30–50% cellulose, 15–35% hemicellulose, and 10–20% lignin depending on particular lignocellulosic materials of interest [9]. Each of these compositions is bound tightly together with covalent and hydrogen bonds [10]. Certainly, like most biomasses, lignocellulosic biomass needs to be pretreated and hydrolyzed in order to obtain ready-to-use sugars for microorganisms [11]. Several inhibitors are released or formed during these procedures, including furadehydes derivatives, weak acids, and phenolic compounds [12, 13]. Therefore, to utilize lignocellulosic biomass as a source of fermentable sugars, cellular tolerance of yeast hosts to such inhibitors is considered one of the desirable traits that would allow high-yielding production.

Several industrial yeasts are sensitive to certain inhibitors from the conversion of lignocellulosic biomass to free sugars and there is no exception for *S. passalidarum*. Though this yeast is capable of producing a high ethanol yield from xylose with the highest ethanol yield of 0.43 g ethanol/g xylose at 30 °C [6], it has been known to be sensitive to a furan derivative, furfural [14]. This essentially results in ethanol production at a sub-optimal level. Hence, in this study, furfural (a by-product from acid hydrolysis of 5-carbon sugars in hemicellulose) [15] is our focused inhibitor.

Furfural concentrations in lignocellulosic hydrolysates typically range from 0.2 to 5.0 g/l, depending on the source of biomass and the applied pretreatment techniques [16, 17]. With regards to toxicity, furfural has been reported to induce prolonged lag phases, increase reactive oxygen species (ROS) accumulation, cause DNA fragmentation, affect mitochondria and vacuoles, create redox imbalances, and inhibit glycolytic pathway enzymes [18–20]. Moreover, from a genetic standpoint, furfural induces random mutations in yeast genomes [15, 21]. Genetic alterations caused by furfural have been previously reported in *Saccharomyces cerevisiae*. In the case of *S. cerevisiae* treated with furfural, whole-genome single nucleotide polymorphism (SNP) microarray and sequencing showed varying levels of genetic alteration, including single-base substitutions, loss of heterozygosity, and chromosomal rearrangements leading to aneuploidy [22]. In another study, yeast cells cultured in medium containing a nonlethal dose of 0.6 g/l furfural exhibited aneuploidy, chromosomal rearrangements (including large deletions and duplications), and loss of heterozygosity (LOH) [23].

Evolutionary engineering is one of the approaches used to develop industrial yeast traits [24]. This approach relies on a combination of induced mutation and the subsequent selection of mutants. Induced mutation introduces genetic diversity, while selection ensures that the population evolves in a direction aligned with the desired traits [25, 26]. Evolutionary engineering can be achieved by several techniques ranging from simple transfer of strain in small-scale shake flasks to the use of bioreactors [27]. Though adaptive laboratory evolution (ALE) has been applied to several studies, especially to *S. cerevisiae*, for developing industrially preferable strains [24, 28, 29], there are few reports on the use of evolutionary engineering in *S. passalidarum* and most of them were conducted in *S. passalidarum* strain NRRL Y-27907 (Table 1). As shown in Table 1, ALE was utilized to enhance *S. passalidarum* for three main purposes: improved glucose-xylose co-fermentation [30–32], increased tolerance to hydrolysate inhibitors [33, 34], and increased fermentation ability [35]. To improve glucose-xylose co-fermentation, UV mutagenesis or genome shuffling was conducted together with ALE. While increasing hydrolysate inhibitors tolerance and fermentation ability, ALE was performed by using lignocellulosic hydrolysate as a stressor, which contained a mixture of inhibitors as a selective stressor. For applying furfural as a single stressor, there was only one research that attempted to improve furfural tolerance in *S. passalidarum* NRRL Y-27907, however, the study used a combination of UV mutagenesis and protoplast fusion instead of using ALE [14] (Table 1).

Although biomass-derived hydrolysates, which contain variable composition, along with multiple stressors [15], provide a more realistic representation of industrial conditions and introduces challenges in ALE experiments, potentially hindering the ability to draw clear conclusions. In the presence of multiple stressors, microbial populations may develop adaptations to a combination of stressors rather than specific responses to individual stressors, causing complicated interpretation of the evolved phenotypes. The synergistic effect from mixture stressors causes more toxicity to the cell [36]. This might lead to a slowdown in the adaptation process compared to using a single stressor. Consequently, applying furfural as a single stressor in ALE processes is interesting because using a single stressor provides a more controlled and focused approach, allowing for a deeper understanding of the mechanisms involved in furfural tolerance and the development of strains with improved characteristics for industrial use.

In this study, a furfural-tolerant strain, *S. passalidarum* AF2.5, was successfully developed through adaptive laboratory evolution using furfural as a single stressor. This strain exhibited tolerance to furfural up to 4.0 g/l and enhanced tolerance to HMF and ethanol. The improved furfural tolerance led to a reduction in the prolonged lag phase, accompanied by lower ROS accumulation and decreased nuclear chromatin diffusion. This study demonstrates the potential of employing 17 rounds of evolutionary engineering with a single stressor as a strategy to cultivate industrially appealing *S. passalidarum*, resulting in significant improvements in furfural tolerance and ethanol production.

**Table 1:** Adaptive laboratory evolution of *S. passalidarum* and other techniques to improve furfural tolerance in *S. passalidarum*

Parental strain	Objective	Adapted strain	Methods*	Outcome	References
*S. passalidarum* NRRL Y-27907= (ATCC MYA-4345, CBS 10155), which is mesophile yeast (T_max_ =40 °C) that presents glucose repression and cannot assimilate L-arabinose [7, 37].	1. Improved co-assimilation of glucose and xylose	Spc3	UV mutagenesis and ALE (2-DOG)	Spc3 showed a slight improvement in glucose and xylose co-fermentation compared to WT, however, the consumption rate of both sugars was slower than WT.	[30]
		X2, X5	Genome shuffling between *S. cerevisiae* and *S. passalidarum* and ALE (YP medium containing 20 g/l xylose at 40 °C)	Under mixed sugars of glucose-xylose condition at 40 °C, X2 and X5 could utilize glucose and xylose faster than WT. Both adapted strains produced ethanol 1.5-fold higher than WT.	[31]
		E7	ALE (wood hydrolysate under O_2_-limiting conditions)	Fermentation ability of E7 in both MHH and SM media was performed. Adapted strain could co-metabolize glucose and xylose at similar rates in both media, however xylose utilization was delayed. Ethanol production in MHH required 21 h longer than that in SM. Ethanol production in MHH was 39 g/l with a yield of 0.34 g/g, which was slightly lower than that in SM (did not compare with WT).	[32]
		AF2	ALE (wood hydrolysate and AFEX corn stover hydrolysate under O_2_-limiting conditions)	Fermentation of AF2 in an AFEX hydrolysate took a long time. Xylose was largely delayed. It was utilized after glucose was nearly finished. Ethanol production was rich to the highest level of 23 g/l with a yield of 0.45 at 7 days (did not compare with WT).	
	2. Increased hydrolysate inhibitors tolerance	A5	ALE (sugarcane hydrolysate)	Adapted strain, A5 was capable of fermenting hydrolysate efficiently, reaching ethanol yield and productivity of 0.404 g/g and 0.357 g/l/h, respectively, while the WT was not able to ferment.	[33]
		mutA4	UV mutagenesis and ALE (acetic acid and *Eucalyptus globulus* auto-hydrolysate)	Adapted strain, mutA4 was tolerant to acetic acid. In presence of 4.5 g/l acetic acid, it produced ethanol volumetric productivity and ethanol yield of 7-fold (0.23 g/l/h) and 2-fold (0.48 g/g) higher than WT, respectively. When *Eucalyptus globulus* auto-hydrolysate was used as a culture medium, mutA4 resisted inhibitors usually found in this hydrolysate and was able to co-ferment glucose, xylose and cellobiose under microaerobic condition without lag phase.	[34]
		FS22 (hybrid strain)	UV mutagenesis (furfural) and protoplast fusion	Hybrid strain, FS22 was able to grow and produce ethanol at a yield of 0.4 g/g in 75% liquid fraction of pretreated wheat straw (WSLQ) medium with addition of 30 g/l xylose.	[14]
	3. Increased fermentation ability	E11	Cell recycling, cell mating and high-throughput screening and ALE (various types of hydrolysates)	Adapted strain, E11 showed a 3-fold increase in specific fermentation rate compared to WT and an ethanol yield greater than 0.45 g/g substrate while co-utilizing cellobiose, glucose and xylose.	[35]
*S. passalidarum* CMUWF1−2, which is thermotolerant yeast (T_max_ = 42 °C that presents no glucose repression and can assimilate L-arabinose [6].	Improved furfural tolerance	AF2.5	ALE (furfural)	Adapted strain, AF2.5 showed improvement of furfural tolerance together with ethanol and HMF tolerances compared with WT, while maintaining the ability of simultaneous utilization of glucose and other sugars.	This study

* Phrase in parentheses represent stressor used in ALE; 2-DOG, 2-deoxy-D-glucose; AFEX, Ammonia Fiber Expansion; MHH, maple hemicellulose hydrolysate; HMF, 5-hydroxymethylfurfuraldehyde; WT, wild type or parental strain

## Methods

### Yeast strains and cultivation media

*Spathaspora passalidarum* CMUWF1−2 isolated from soil in Mae Taeng district, Chiang Mai province, Thailand [6] was used in this study. This yeast strain was cultured on yeast extract-peptone-dextrose (YPD) agar (10 g/l yeast extract, 20 g/l peptone, 20 g/l dextrose, and 15 g/l agar) and incubated at 30 °C for 2 days. A single colony from the agar plate was transferred into YPD broth (10 g/l yeast extract, 20 g/l peptone, and 20 g/l dextrose) for subsequent experiments. All yeast strains in this study were kept in YPD broth with 25% (v/v) glycerol at -20 °C for long-term storage.

### Spot test analysis

For testing stress tolerance, a spot test was performed following the method described by Rodrussamee et al. [6]. After washing the cells, the suspended cells (1 × 10^7^ cells/ml) were 10-fold sequentially diluted to 10^− 4^. Five microliters of each dilution were spotted onto an agar plate in the presence and absence of chemicals of interest as specified in the relevant sections.

### Adaptive laboratory evolution (ALE) of CMUWF1−2 for enhancing furfural tolerance

Before enhancing furfural tolerance in *S. passalidarum* CMUWF1**−**2, tolerance of CMUWF1**−**2 to furfural (FF) was investigated via spot test. Cell was spotted onto YPD agar supplemented with different concentrations of FF at 0.0, 1.0, 1.5, 2.0, 2.5, 3.0, 3.5, and 4.0 g/l. The plates were then incubated at 30 °C for 48 h or 72 h.

**Fig. 1:**
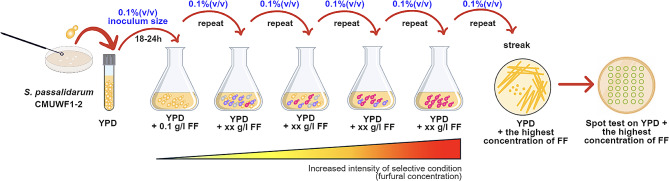
Schematic overview of adaptive laboratory evolution (ALE) via serial transfer in shake flasks using FF as a stress inducer

In this study, ALE via serial transfer in shake flasks was performed for improving FF tolerance in CMUWF1**−**2 as a following procedure (Fig. 1). Yeast cells gradually reduce FF to less toxic furfuryl alcohol during fermentation, causing a prolonged lag phase in the initial stage of incubation. Once this inhibitor is reduced, growth resumes [17]. Thus, the number of subcultures for each concentration of FF depended on the growth rate of each round. To be more specific, if cells were able to reduce a prolonged lag phase and enter the exponential phase more efficiently than the previous round at the same concentration, it suggests that the cells have adapted themselves and are relatively ready for reinoculation into a higher concentration of the inducer. Cell was precultured in 5 ml of YPD broth and incubated at 30 °C with shaking at 150 rpm for 18–24 h. One hundred microliters of preculture broth were then inoculated into 100 ml of fresh YPD broth [0.1% (v/v) inoculum size] supplemented with an initial FF concentration of 1.0 g/l in 250 ml-Erlenmeyer flask and incubated at 30 °C with shaking at 150 rpm.

At each concentration of FF, the yeast was reinoculated at 0.1% into fresh media supplemented with the same or increased FF concentrations. The initial OD_600_ before starting each round was approximately OD_600_ = 0.01–0.05. The cells were cultured until the culture media became turbid. After measuring, when the culture media reached OD_600_ = 10–15, it was assumed that the cells were in an exponential phase, as demonstrated in a previous publication on the growth of CMUWF1–2 cultured in YPD broth at 30 °C [6]. At that concentration, after subculturing until the duration of the lag phase from the previous round was reduced and stabilized, cells were transferred to an elevated concentration, anticipated to increase by 0.5 g/l from the previous one. However, if it was observed that cell growth was inadequate at the transferred concentration, the cells would be transferred to a lower concentration. Eventually, the strain was transferred to the final concentration of FF, which was the highest concentration this strain could tolerate. Subsequently, one full loop of culture media was restreaked on YPD agar with that highest concentration. After incubation, five single colonies were randomly selected. These colonies were then spotted on YPD agar with that concentration, and only one adapted strain, which exhibited the highest growth, was chosen for further experiments.

### Species identification

After obtaining the adapted strains from the ALE process, nucleotide sequencing of the D1/D2 domain from the large subunit (LSU) rRNA gene was conducted to verify for confirming that such observations were not a result of unintentional contaminations. Genomic DNA extraction was performed as described previously [38]. The forward primer NL-1 (5′-GCATATCAATAAGCGGAGGAAAAG-3′) and reverse primer NL-4 (5′-GGTCCGTGTTTCAAGACGG-3′) were utilized to generate the PCR product of the D1/D2 domain [39]. The PCR product was verified by agarose gel electrophoresis and purified by using a GF-1 AmbiClean Kit (Vivantis, Malaysia). The sequences of the PCR product were determined by 1st BASE (Selangor, Malaysia) and subjected to a BLAST homology search [40]. The determined nucleotide sequence was deposited in the DNA Data Bank of Japan (DDBJ).

### Characterization of furfural-adapted strain on stress tolerance and glucose repression

The obtained furfural-adapted strain was investigated for its other stress tolerances and its glucose repression compared with the wild-type strain (CMUWF1**−**2). For testing the tolerance of high concentrations of glucose, a spot test was carried out on YP agar (YP; 10 g/l yeast extract, 20 g/l peptone, and 15 g/l agar), while tolerances of high concentration of ethanol (EtOH) and 5-hydroxymethylfurfuraldehyde (HMF), YPD agar was used. For investigating tolerances to high concentrations of glucose, EtOH, and HMF, YP or YPD agar was supplemented with various concentrations of glucose (25, 30, 35, and 40% (w/v)), EtOH (6, 7, 8, 10, and 12% (v/v)), and HMF (1.0, 1.5, 2.0, and 2.5 g/l), respectively. For testing high-temperature tolerance, cells were spotted on YPD agar, and then incubated at various temperatures; 30, 37, 40, and 42 °C. For testing glucose repression, cells were spotted onto YP agar containing 0.01% (w/v) of a glucose analog (2**−**deoxyglucose; 2**−**DOG and 20 g/l sugars as follows; glucose (Glu), mannose (Man), galactose (Gal), arabinose (Ara), and xylose (Xyl). The concentration of 0.01% (w/v) 2–DOG was selected because, at this concentration, it could demonstrate glucose repression on xylose and galactose in *Kluyveromyces marxianus*, serving as a positive control, while not affecting CMUWF1–2 [6].

### Effects of furfural on growth, xylose utilization, and ethanol production of furfural-adapted strain compared with CMUWF1−2

YPXyl (10 g/l yeast extract, 20 g/l peptone, and 20 g/l xylose), YPXyl supplemented with 2.0 g/l FF, and YPXyl supplemented with 3.0 g/l FF were used as fermentation media to evaluate the effect of FF on growth, xylose utilization and ethanol production of the adapted strain in parallel with its respective wild type. Cells were precultured in 5 ml of YPXyl broth incubated in shaking condition at 30 °C with shaking at 150 rpm for 18–24 h. The culture broth of 0.1% (v/v) inoculum was then inoculated into 20 ml of fresh YPXyl media in 100-ml Erlenmeyer flasks. After the incubation in the same condition, culture broth of 0.1% (v/v) inoculum was inoculated into 50 ml of fermentation media in 125-ml Erlenmeyer flasks with the initial OD_600_ = 0.05. During cultivation at 30 °C with shaking at 150 rpm for 96 h, each sample was taken every 12 h. Growth was observed by measuring optical density at 600 nm. Xylose utilization and ethanol production were analyzed via High-Performance Liquid Chromatography (HPLC) (Shimadzu Corporation, LC-20 A Series, Japan) equipped with an Aminex HPX-87 H column (Bio-Rad Laboratories, Inc.) and a refractive index (RI) detector running at 40 °C with 5 mM H_2_SO_4_ as eluent at a flow rate of 0.80 ml/min. To quantify xylose and ethanol in the samples, serial dilutions of commercial standards, xylose (Himedia, India) and ethanol (AnalaR NORMAPUR, VWR Chemicals), were used to prepare calibration curves.

### Reactive oxygen species analysis

*Spathaspora passalidarum* CMUWF1**−**2 and the adapted strain were precultured in 50 ml of YPD broth at 30 °C with shaking at 150 rpm for 18−24 h. The cells were then inoculated with an initial at OD_600_ = 5 into 3 ml of YPD, YPD supplemented with 2.0 g/l FF, and YPD supplemented with 70 mM hydrogen peroxide (H_2_O_2_) in 16 × 100 mm sterile test tubes and further incubated at the same condition for 24 h. Cells at 0 and 24 h were harvested by adjusting cell concentration as OD_600_ = 5 by using 10 mM potassium phosphate buffer (PPB; pH 6.8). Cells were washed 3 times with 10 mM PPB (pH 6.8) and resuspended in 500 µl of the same buffer. Cells were stained with 2′, 7′-dichlorofluorescein diacetate (H_2_DCFDA) (Sigma-Aldrich, USA) at a final concentration of 10 µM for 1 h before harvesting. Cells were then washed 3 times with 10 mM PPB (pH 6.8) and resuspended in 500 µl of the same buffer. Cells were disrupted with glass beads. The supernatants after cell disruption were collected by centrifugation at 14,000 rpm for 10 min, 4 °C, and then used for reactive oxygen species (ROS) analysis. ROS inside the cells was measured by detecting DCF fluorescence intensity via molecular devices SpectraMax i3x plate reader (excitation, 504 nm, and emission, 524 nm) and the protein concentration by Lowry method [41, 42]. Fluorescence intensity was normalized to the protein concentration of each sample. Relative fluorescence was determined by dividing the normalized value of each condition after 24 h of incubation by the corresponding normalized value of the same condition at 0 h.

### Nuclear chromatin morphology

Single colonies of *S. passalidarum* CMUWF1**−**2 and the adapted strain were inoculated in 5 ml of YPD broth as seed cultures. The seed cultures were then inoculated and cultured in YPD and YPD supplemented with 2.0 g/l FF at 30 °C with shaking at 150 rpm. During cultivation, the samples were collected at 0, 24, and 36 h and then adjusted to be obtained OD_600_ = 0.2 using deionized water (DIW). The OD_600_-adjusted cells were collected by centrifugation and washed one time by DIW before resuspending in 10 µl of DIW and 190 µl of absolute ethanol (RCI Labscan Ltd., Thailand). These cells were then stained with 1 µl of 2 mg/ml diaminophenylindole (DAPI) (Sigma-Aldrich, USA), gently mixed, and incubated in the dark at room temperature for 10 min. The collected cells were washed with 200 µl of DIW three times and then resuspended in 20 µl of DIW. This suspension was dropped on an adhesion slide (Scientific Co., Ltd, USA) and observed under a fluorescence microscope with an ultraviolet filter (Nikon, Japan) [19]. The percentage of cells with diffuse chromatins was calculated as follows.

$$\begin{aligned} & {\text{Cells}}\,{\text{with}}\,{\text{diffuse}}\,{\text{chromatin}}\,\left( \% \right)\,= \\ & \frac{{{\text{Number}}\,{\text{of}}\,{\text{cells}}\,{\text{with}}\,{\text{diffuse}}\,{\text{chromatin}} \times 100}}{{{\text{Total}}\,{\text{number}}\,{\text{of}}\,{\text{cells}}}} \\ \end{aligned}$$

### Statistical analysis

To determine significant differences between treatments of nuclear chromatin morphology analysis and ROS analysis, One-way analysis of variance (One-way ANOVA) via Tukey′s test and paired sample t-test were used, respectively. Both statistical tests were analyzed with *p* < 0.05.

## Results

### Adaptive laboratory evolution of CMUWF1−2 for enhancing furfural tolerance

**Fig. 2:**
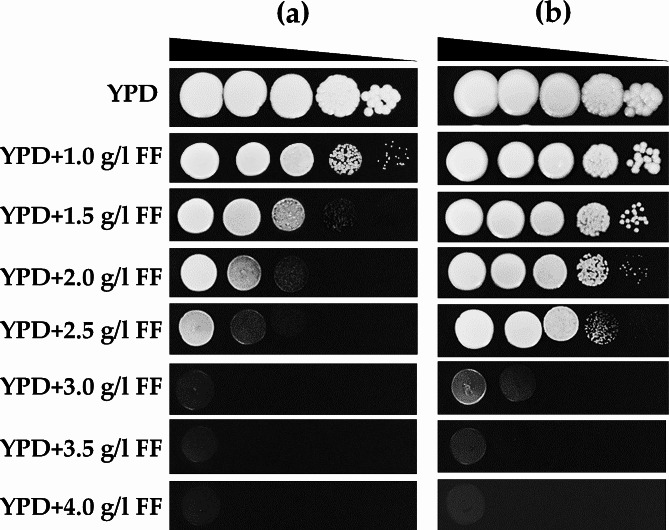
The level of FF tolerance in *S. passalidarum* CMUWF1−2 incubated for 48 h (**a**) and 72 h (**b**) at 30 °C. Various cell concentrations of CMUWF1−2 were spotted on YPD agar supplemented with 0.0, 1.0, 1.5, 2.0, 2.5, 3.0, 3.5 and 4.0 g/l FF. The cell suspension, containing approximately l × 10^7^ cells/ml was 10-fold serially diluted and spotted onto agar plates. Data were reproduced by two independent experiments

The furfural (FF) tolerance level in the wild type, *S. passalidarum* CMUWF1−2, was initially assessed by spotting various cell concentrations on YPD agar supplemented with 0.0–4.0 g/l FF. When comparing cell growth at different incubation times, 48 and 72 h, it was evident that the growth of cells incubated for 72 h was better than that of cells incubated for 48 h (Fig. 2ab).

Incubation for 48 h in the presence of 1.0 g/l FF showed that CMUWF1−2 could grow at every cell concentration; however, the size of the cells decreased (Fig. 2a). As the concentration of FF gradually increased beyond 1.0 g/l, cell growth was dramatically reduced under both incubation times, especially during the 48-h incubation (Fig. 2a). The highest concentration of FF at which CMUWF1−2 hardly grew was 3.0 g/l, even after further incubation for 72 h. Meanwhile, at 3.5 and 4.0 g/l FF, the growth of CMUWF1−2 after both 48 and 72 h of incubation was completely inhibited (Fig. 2ab). Based on this result, it was found that FF had an effect on CMUWF1–2 beginning at 1.0 g/l FF.

**Fig. 3:**
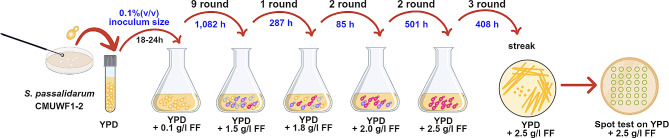
Flow diagram of adaptive laboratory evolution for obtaining AF2.5

Evolutionary engineering through the ALE approach was conducted to enhance the ability of CMUWF1−2 to tolerate FF (Fig. 3). Before commencing ALE, determining the suitable initial FF concentration, which would inhibit yeast cells to a certain extent without causing severe damage, is crucial. Our preliminary results indicated that 1.0 g/l FF was a suitable starting point for evolution. Consequently, CMUWF1−2 was initially inoculated in YPD broth supplemented with 1.0 g/l FF. Once the inoculum, representing 0.1% of cells cultured in each round, grew to OD_600_ = 10–15, they were transferred to the subsequent round. At 1.0 g/l FF, cells were cultured for 9 rounds (1,082 h). Subsequently, the cells underwent sequential subculture with gradually increased concentrations: 1.5, 1.8, 2.0, and 2.5 g/l FF, with the passage repeated for 1, 2, 2, and 3 rounds, respectively. After a total of 17 rounds of cultivation for 2,715 h from sequential subculture in an initial concentration of 1.0 g/l FF until reached the final concentration of 2.5 g/l FF, one full loop of culture was streaked on YPD medium containing 2.5 g/l FF. After incubation, five single colonies of adapted strains (No.1-No.5) were randomly selected to be spotted on YPD agar containing 2.5 g/l FF. The results indicated that all adapted strains exhibited higher tolerance to 2.5 g/l FF compared to the wild type (Additional file 2: Fig. S2). However, all adapted strains demonstrated a similar level of tolerance to FF. Consequently, only one of the five adapted strains, namely AF2.5, was selected for further experiments. Attempts to subculture the wild type at concentrations higher than 2.5 g/l still did not yield mutants superior to AF2.5.

**Fig. 4:**
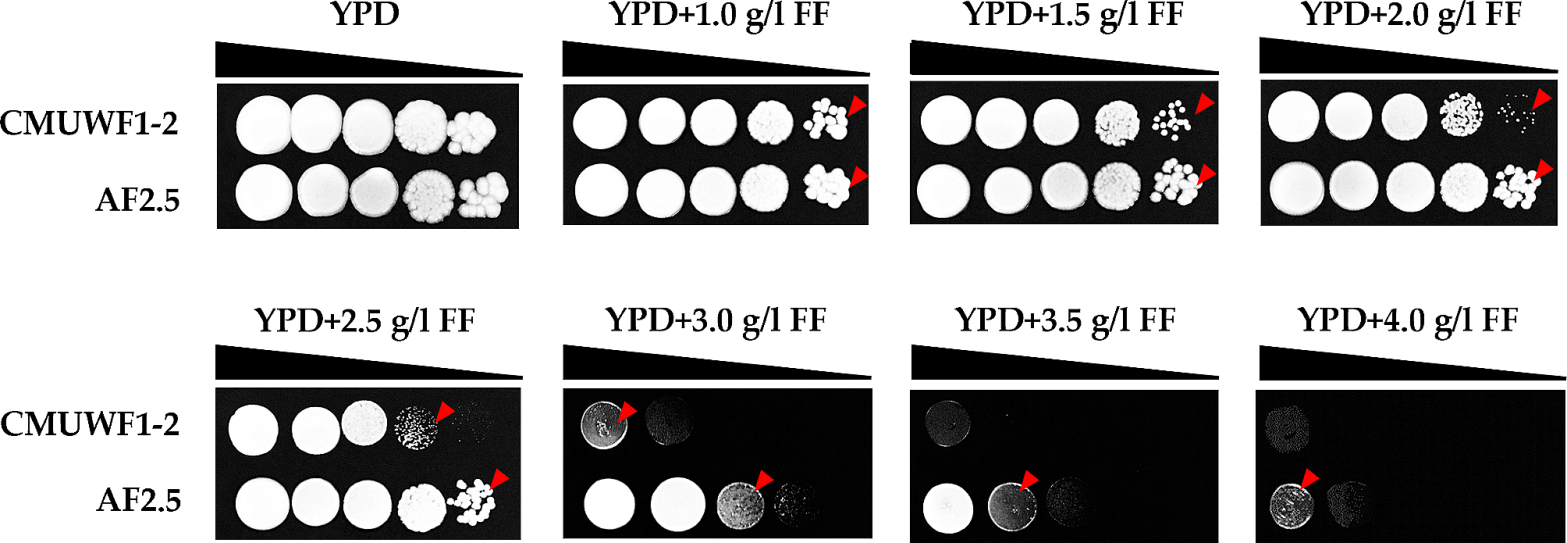
Growth of *S. passalidarum* CMUWF1−2 and AF2.5 on YPD agar supplemented with different concentrations of FF. Both strains were spotted on YPD agar supplemented with 0.0, 1.0, 1.5, 2.0, 2.5, 3.0, 3.5, and 4.0 g/l FF. The cell suspension, containing approximately l × 10^7^ cells/ml was 10-fold serially diluted and spotted onto agar plates, which were incubated at 30 °C for 72 h. Data were reproduced by two independent experiments. Arrows indicate the presence of spotted cells

In the presence of FF ranging from 1.0 g/l to 2.0 g/l, the spotted cell concentrations at every dilution (10^− 1^ to 10^− 4^) of both CMUWF1–2 and AF2.5 were observed (Fig. 4). However, the colony size decreased with an increase in FF concentration, particularly noticeable in cells spotted at a dilution of 10^− 4^. FF tolerance became evident when CMUWF1−2 and AF2.5 were grown in the presence of 2.5 g/l FF. The spotted cell concentration at a dilution of 10^− 3^ was faint for CMUWF1–2, while at a dilution of 10^− 4^, AF2.5 exhibited intense growth. At concentrations higher than 2.5 g/l FF, AF2.5 continued to demonstrate enhanced growth compared with the wild type, indicating the success of ALE. Interestingly, AF2.5 could grow at concentrations up to 4.0 g/l, while CMUWF1–2 was completely inhibited in the presence of FF exceeding 3.0 g/l (Fig. 4). To verify that such observations were not a result of unintentional contaminations, species identification of AF2.5 was conducted by sequencing the D1/D2 domain from the large-subunit (LSU) of the ribosomal DNA gene [6, 43, 44]. The sequencing result confirmed that the adapted strain AF2.5 was *S. passalidarum*. The determined nucleotide sequence was deposited in the DNA Data Bank of Japan (DDBJ) as LC683705.

### Characterization of furfural-adapted strain, AF2.5 on stress tolerance and glucose repression

**Fig. 5:**
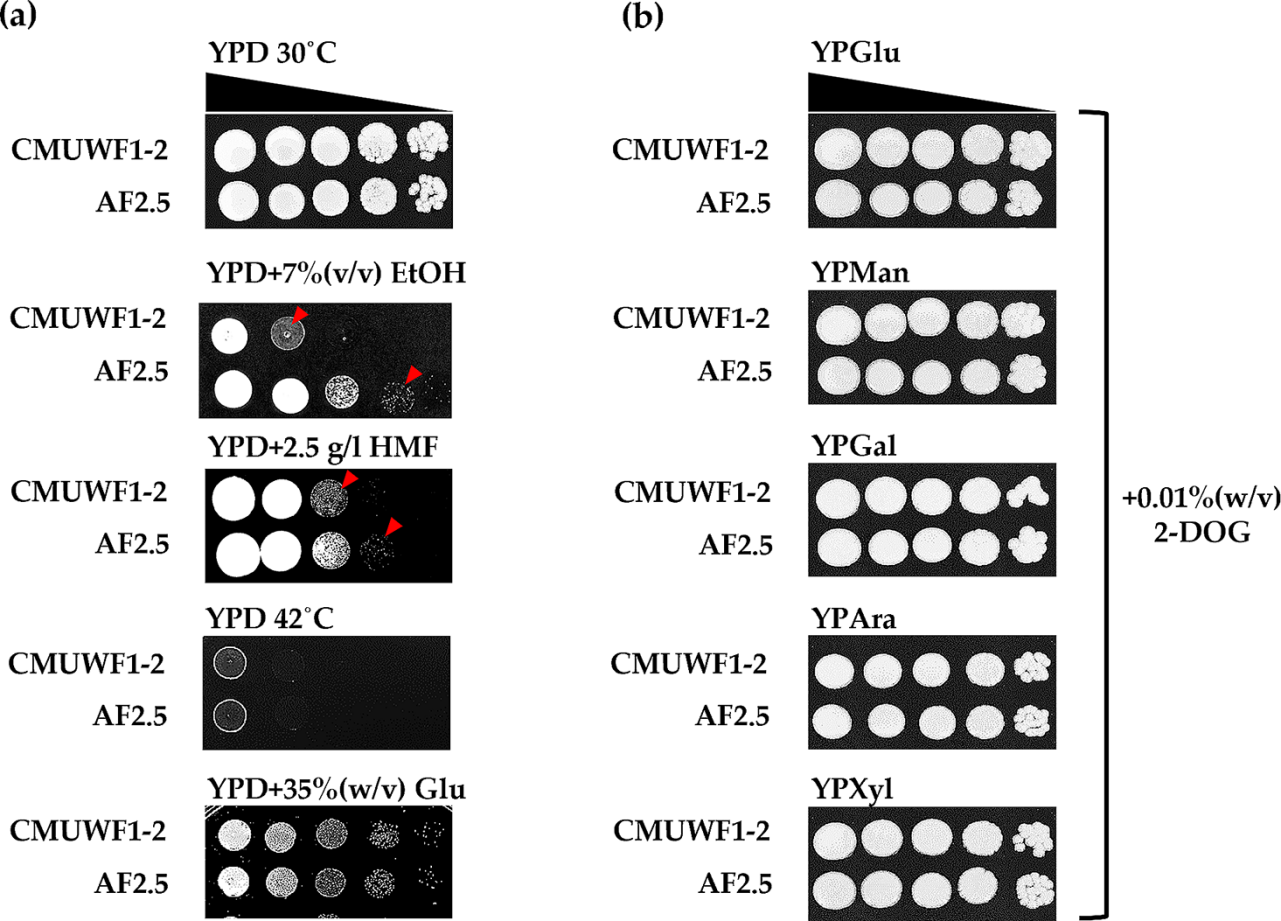
Comparison of stress tolerances (**a**) and glucose repression (**b**) between *S. passalidarum* CMUWF1−2 and AF2.5. The strains were spotted on YPD agar under different stresses and YP with various sugars supplemented with 2–DOG to observe stress tolerances and glucose repression, respectively. The cell suspension, containing approximately l × 10^7^ cells/ml was 10-fold serially diluted and spotted onto agar plates, which were incubated for 72 h. Data were reproduced by two independent experiments. Arrows indicate the presence of spotted cells

*Spathaspora passalidarum* CMUWF1−2 has been reported to tolerate high concentrations of glucose and exhibit some level of tolerance to ethanol. It effectively ferments all monosaccharides in lignocellulosic hydrolysates except arabinose at elevated temperatures (up to 40 °C) with no glucose repression [6]. Therefore, strain AF2.5 was assessed for its tolerance levels to high concentration of glucose, ethanol, elevated temperature, and, additionally, the furan derivative, HMF. Furthermore, its susceptibility to glucose repression was compared with that of its respective parental strain. The concentrations selected for demonstration in Fig. 5a were the highest concentrations at which CMUWF1–2 could grow. The strain AF2.5 was found to tolerate EtOH and HMF at higher levels than the wild type, as it could grow better at 7% (v/v) ethanol and 2.5 g/l HMF, respectively (Fig. 5a and Additional file 1: Fig. S1). However, both of these strains showed similar tolerance levels to glucose [35% (w/v)] and the elevated temperature (42 °C), as shown in Fig. 5a. Importantly, the adapted strain could maintain the parental ability to utilize glucose simultaneously with other sugars as it could grow well on all YP agar with various sugars and 0.01% (w/v) 2–deoxyglucose (2–DOG) [6] (Fig. 5b).

### Effects of furfural on growth, xylose utilization, and ethanol production of AF2.5 compared with CMUWF1*−*2

**Fig. 6:**
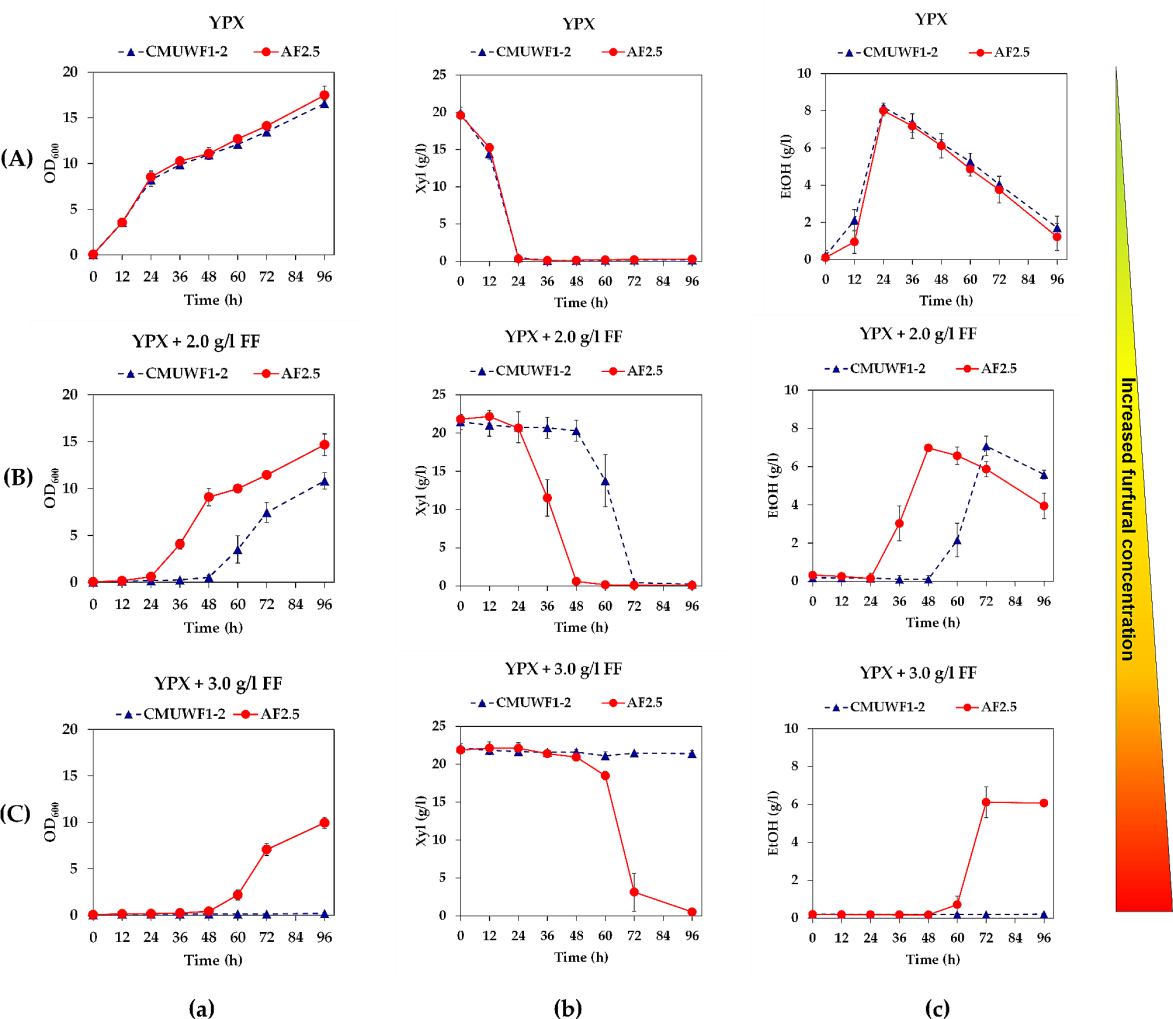
Effects of FF on growth **(a)**, xylose utilization **(b)**, and ethanol production **(c)** of *S. passalidarum* CMUWF1−2 and AF2.5 in YPXyl broth with 0.0 g/l **(A)**, 2.0 g/l **(B)** and 3.0 g/l **(C)** FF. Symbols for the data are as follows:, CMUWF1–2;, AF2.5. All data are average of 3 replicates and error bars represent standard deviations

To evaluate the effect of FF on growth, xylose utilization, and ethanol production of *S. passalidarum* CMUWF1−2 and AF2.5, they were cultivated in YPXyl broth with and without 2.0 and 3.0 g/l FF. There are no significant differences in growth, xylose consumption, and ethanol when these strains were cultured in the absence of FF (Fig. 6A; Table 2). At 2.0 g/l FF, the values for all parameters (Max.µ_x/s_, Max.γ_s_, Max.EtOH, Max.Y_p/s_) of both CMUWF1–2 and AF2.5 showed no statistically significant differences (Table 2). However, AF2.5 exhibited enhanced performance, demonstrating faster growth, sugar utilization, ethanol production, and ethanol yield compared to CMUWF1−2 (Fig. 6B). At 3.0 g/l FF, only AF2.5 exhibited growth, xylose utilization, and ethanol production (Fig. 6C).

When comparing the growth of both strains, it was found that in the presence of 2.0 g/l FF, they showed an extended lag phase in comparison with the treatments without FF (Fig. 6aB) suggesting that FF at this concentration could affect the growth of both strains. Noticeably, AF2.5 entered the log phase after 24 h of incubation, while CMUWF1−2 entered the log phase after 48 h of incubation with the maximum growth rate of 0.42±0.09 at 48 h and 0.33±0.06 at 72 h, respectively (Table 2). Even more, it showed that AF2.5 could grow in YPXyl with 3.0 g/l FF where the growth of CMUWF1−2 was completely inhibited (Fig. 6aC). This emphasizes improvements in FF tolerance in the growth of the adapted strain.

Xylose utilization and ethanol production in the presence of FF of CMUWF1−2 and AF2.5 were also slower than when cultured in the absence of FF. Moreover, it was more evident when the concentration of FF was increased. Certainly, in the presence of FF, AF2.5 was able to consume xylose and produce the highest amount of ethanol faster than CMUWF1−2 (Fig. 6b and c (B, C). When AF2.5 was cultured in YPXyl with 2.0 g/l FF, it could consume all available xylose and produced the highest EtOH yield at 0.33 ± 0.01 g EtOH/g xylose within 48 h (Table 2), while CMUWF1−2 took 72 h to perform the same task (Fig. 6bB and 6cB). Furthermore, at 3.0 g/l FF, complete xylose consumption was observed from AF2.5 after 96 h of incubation (Fig. 6bC), and the highest EtOH yield was produced at 0.33 ± 0.01 g EtOH/g xylose within 72 h (Fig. 6cC) (Table 2). As mentioned, CMUWF1−2 could not grow at this concentration of FF and, as expected, it did not utilize xylose and produce ethanol (Fig. 6abc (C)).

**Table 2:** Growth, xylose utilization, and ethanol yield from *S. passalidarum* CMUWF1−2 and AF2.5 cultivated in YPXyl broth with 0.0, 2.0, and 3.0 g/l FF

Parameters	FF conc. (g/l)	CMUWF1–2	AF2.5
**Max. µ**_**x/s**_ **(h** ^**− 1**^ **)**	0.0	0.39(24) ± 0.05^a^	0.42(24) ± 0.04^a^
	2.0	0.33(72) ± 0.06^a^	0.42(48) ± 0.09^a^
	3.0	0.00 ± 0.00^a^	0.41(72) ± 0.01^b^
**Max. γ**_**s**_ **(g/l.h** ^**− 1**^ **)**	0.0	1.16(24) ± 0.07^a^	1.24(24) ± 0.05^a^
	2.0	1.11(72) ± 0.28^a^	0.91(48) ± 0.19^a^
	3.0	0.00 ± 0.00^a^	1.28(72) ± 0.17^b^
**Max. EtOH (g/l)**	0.0	8.18(24) ± 0.23^a^	8.01(24) ± 0.28^a^
	2.0	7.08(72) ± 0.52^a^	6.98(48) ± 0.14^a^
	3.0	0.00 ± 0.00^a^	6.12(72) ± 0.81^b^
**Max. Y**_**p/s**_ **(g/g)**	0.0	0.42(24) ± 0.02^a^	0.41(24) ± 0.01^a^
	2.0	0.34(72) ± 0.01^a^	0.33(48) ± 0.01^a^
	3.0	0.00 ± 0.00^a^	0.33(72) ± 0.01^b^

Max.µ_x/s_, Maximum growth rate; Max.γ_s_, Maximum sugar utilization rate; Max.EtOH, Maximum EtOH production; Max.Y_p/s_, Maximum ethanol yield

Values in parentheses represent cultivation times that are required for reaching the maximum values; ±, SD from three independent experiments; Different letters represent significant statistical differences between CMUWF1*−*2 and AF2.5 of each parameter in the same FF concentration according to Student’s t-tests with *p* < 0.05

### Effects of furfural on reactive oxygen species accumulation

**Fig. 7:**
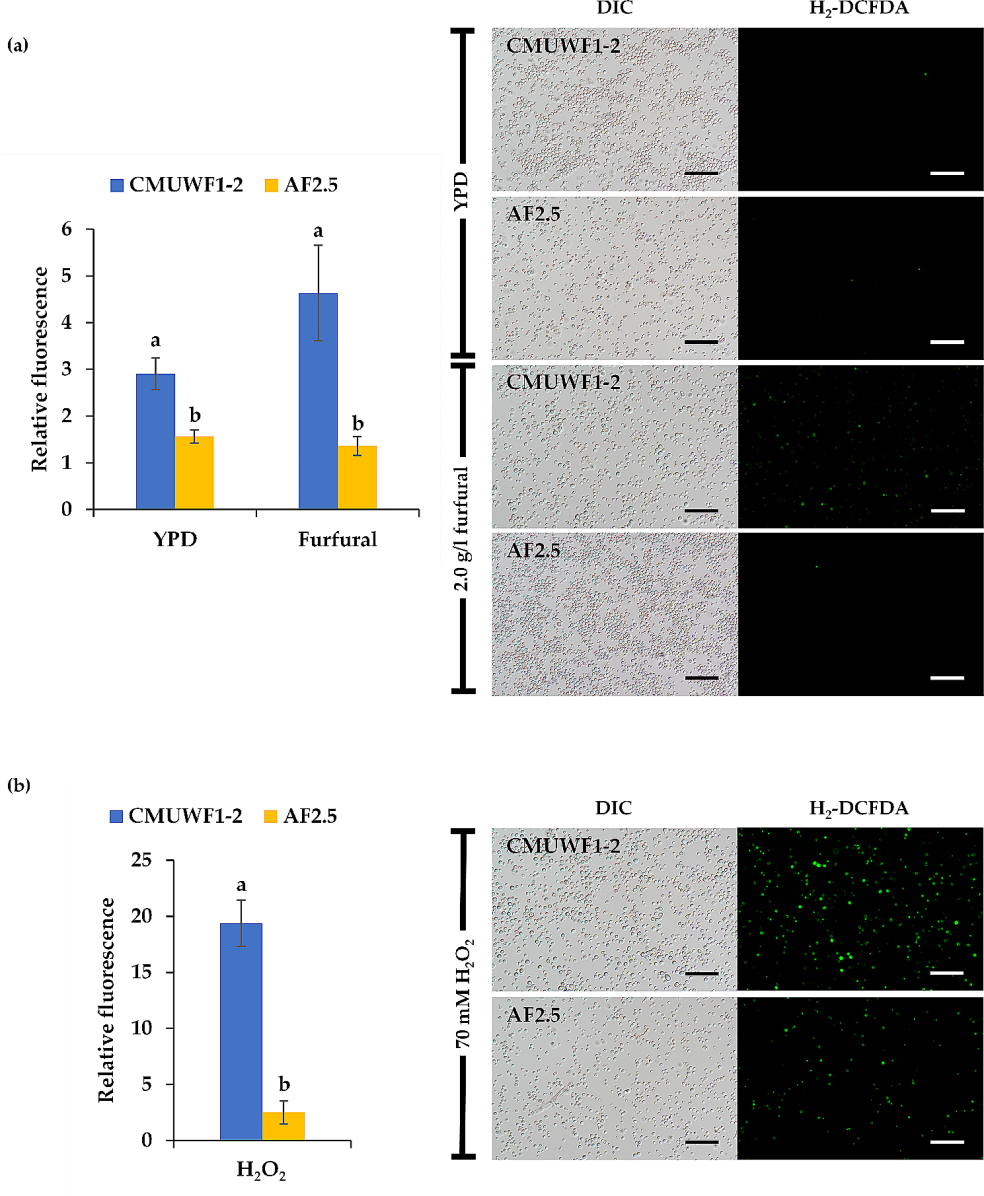
The intracellular ROS level of *S. passalidarum* CMUWF1−2 and AF2.5 cells when cultivated in YPD broth with and without 2.0 g/l FF (**a**) and YPD broth with 70 mM hydrogen peroxide (H_2_O_2_) (**b**) incubated for 24 h. Different letters indicate statistically significant differences between CMUWF1−2 and AF2.5 in each condition, which were analyzed by pair sample t-test (*p* < 0.05). All data are averages of three replicates and error bars represent standard deviations. Black and white bars represent 50 μm

Prior studies in *S. cerevisiae* have demonstrated that FF can induce the generation of reactive oxygen species (ROS) [19, 45, 46]. To examine the impact of FF on ROS accumulation in *S. passalidarum*, both CMUWF1–2 and AF2.5 strains were stained with 2´,7´-dichlorofluorescein diacetate (H_2_DCFDA), and the production of ROS was quantified based on fluorescence intensity. Yeast cells were treated with and without 2.0 g/l FF for 24 h. Moreover, the positive control for detecting ROS was the presence of hydrogen peroxide (H_2_O_2_). The results were significant in that for both YPD and YPD with 2.0 g/l FF, the ROS accumulation level in CMUWF1−2 was higher than in AF2.5 by 1.86 and 3.41 times, respectively (Fig. 7a). Furthermore, in YPD with H_2_O_2_ medium, CMUWF1−2 exhibited a ROS level 7.72 times higher than that of AF2.5 (Fig. 7b).

### Effects of furfural on nuclear chromatin morphology

The previous studies in *S. cerevisiae* showed that FF could induce ROS [19, 45, 46], which consequent cellular damages including diffuse nuclear chromatins. Thus, in this study, nuclear morphology was used as an indicator to investigate the effects of FF as a cause of cellular oxidative stress in *S. passalidarum*. Strains CMUWF1−2 and AF2.5 were cultivated in YPD with 2.0 g/l FF compared with the treatment without FF addition. The nuclear chromatins were observed at various time points (0, 24, and 36 h). Typically, yeast nuclei tend to be tightly compacted in stable stages and diffuse in the multiplying or adapting stages [19, 47].

**Fig. 8:**
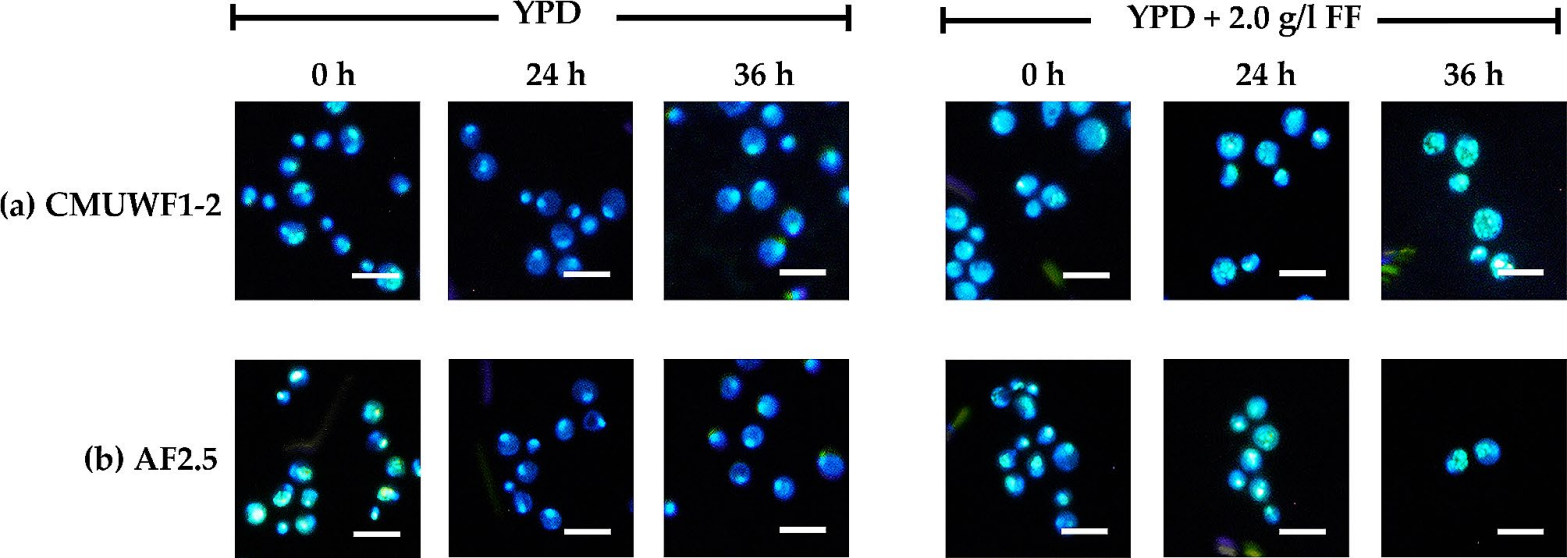
Nuclear chromatins morphology of *S. passalidarum* CMUWF1−2 (**a**) and AF2.5 (**b**) in YPD with and without 2.0 g/l FF. Cells were cultured in YPD and YPD + 2.0 g/l FF at 30*°*C, 150 rpm. After cultivation, cells at 0, 24, and 36 h were collected to stain with diaminophenylindole (DAPI) and observed under a fluorescent microscope. White bars represent 10 μm

**Fig. 9:**
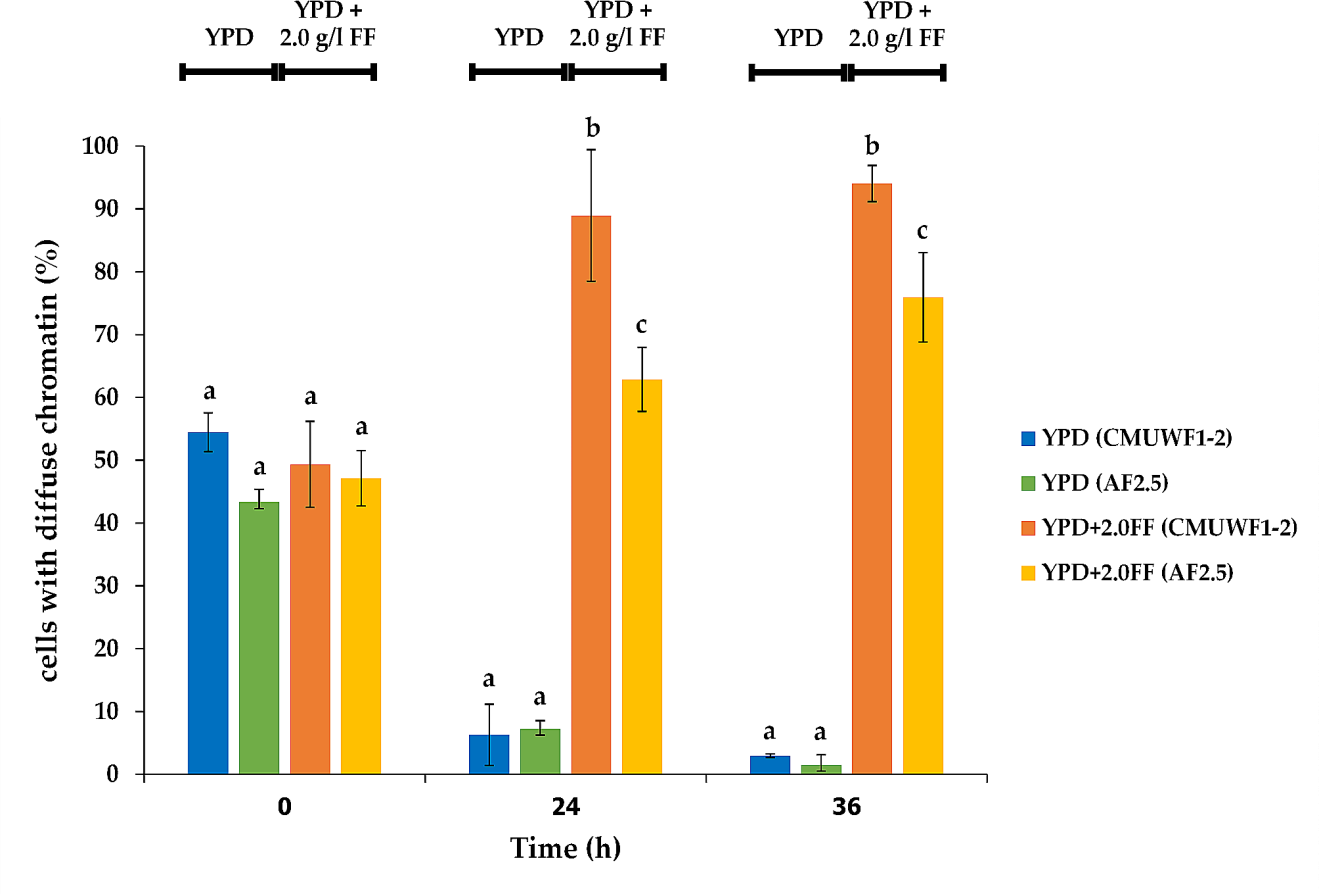
Percentage of cells with diffuse chromatins of *S. passalidarum* CMUWF1−2 and AF2.5 when cultivated in YPD broth with and without 2.0 g/l FF for 0, 24, and 36 h. Different letters indicate statistically significant differences between treatments in each time point (*p* < 0.05), which were analyzed by One-way ANOVA and Tukey′s tests. All data are averages of three replicates and error bars represent standard deviations

At 0 h with the absence and presence of FF, no significant difference in regards to cells with diffuse chromatins was observed in CMUWF1−2 and AF2.5 (Figs. 8 and 9). In the absence of FF at 0 h, the numbers of cells with diffuse nuclear chromatins of CMUWF1−2 and AF2.5 were 54.45±3.07% and 43.32±2.04%, respectively. Nevertheless, the number of both cells with diffuse nuclear chromatins decreased when cells were incubated without FF for a longer period (Fig. 9). In the presence of FF at 0 h, the numbers of cells with diffuse nuclear chromatins of CMUWF1−2 and AF2.5 were 49.34±6.85% and 47.12±4.41%, respectively, which were similar to the treatment without FF (Fig. 9). However, the trends were opposite as after incubation for 24 and 36 h, the number of cells with diffuse nuclear chromatins increased in both strains (Figs. 8 and 9) confirming that FF addition contributed to nuclear chromatin diffusion. When comparing CMUWF1−2 and AF2.5 in the presence of FF at 24 h, diffusion of nuclear chromatin was 88.94±10.48% and 62.86±5.10%, respectively. Similar to the result in the presence of FF at 36 h, where the values were 94.04±2.87% and 75.94±7.11% for CMUWF1−2 and AF2.5, respectively (Fig. 9). Overall, these results showed that the numbers of diffuse chromatins in AF2.5 were 1.41 and 1.24 times less than in wild type at 24 and 36 h, respectively when cultivated in the presence of 2.0 g/l FF.

## Discussion

Adaptive laboratory evolution (ALE) used for increasing hydrolysate inhibitor tolerance and enhancing fermentation performance in the *S. passalidarum* type strain (NRRL Y-27907) is typically conducted by exposing the strain to lignocellulosic hydrolysate as a mixture stressor. There was only one study that attempted to enhance furfural (FF) tolerance by employing a combination of UV mutagenesis and protoplast fusion instead of using ALE [14] (Table 1). In this aforementioned study, UV mutagenesis utilizing FF as a single stressor was initially applied to obtain furfural-tolerant mutants, denoted as M7. Compared with the wild type, the mutant exhibited superior growth and achieved higher values for xylose consumption, final ethanol concentration, and ethanol yield, with increases of 1.51, 1.48, and 1.05 folds, respectively, in a synthetic xylose medium containing 2.0 g/l FF. Nevertheless, on a medium containing 75% liquid fraction of pretreatment wheat straw (WSLQ), which included FF and several other inhibitors, this mutant was unable to grow. Moreover, M7 was conducted to protoplast fusion with *S. cerevisiae* ATCC 96581. The hybrid strain, designated as FS22, acquired the desired phenotypes from both parents including the ability for xylose fermentation and tolerance to inhibitors from *S. passalidarum* and *S. cerevisiae*, respectively. As a result, FS22 showed improved performance in 75% WSLQ medium better than parental strain M7 [14]. In this study, ALE was employed to enhance FF tolerance in *S. passalidarum* CMUWF1–2. After transferring for 17 rounds, we successfully obtained a tolerant strain, namely AF2.5. This adapted strain showed enhanced growth at concentrations of more than 1.0 g/l FF compared to the wild type and could grow up to 4.0 g/l FF. Moreover, it was also found to be more resistant to FF than a UV-induced mutant, *S. passalidarum* M7, which could tolerate only 2.0 g/l FF [14].

In addition, AF2.5 demonstrated tolerance to higher concentrations of HMF (2.5 g/l) and ethanol (7% v/v) compared to CMUWF1–2. It was observed that the enhanced FF tolerance in AF2.5 corresponded to increased tolerance to HMF. Since HMF is another furan derivative formed through the acid-catalyzed dehydration of hexoses, primarily fructose and glucose, the modes of toxicity share similarities with FF [12, 15]. Thus, the mechanisms of FF and HMF tolerance in yeast may share similarities. Previously reported works in *S. cerevisiae* and *Candida tropicalis* suggested that multiple genes may be involved in yeast FF and HMF tolerances such as those related to pentose phosphate pathway and transporters [15, 48–53]. Though, in this study, we have yet to identify causal mutations from the ALE, we hypothesize that these phenotypic modifications could be results from changes in gene expression levels, damages and mutations induced by FF [15]. It has been reported that alterations of genes encoding enzymes in pentose phosphate pathway (PPP) such as *ZWF1*, *GND1*, *RPE1*, and *TKL1* were involved in FF tolerance. To give an example, when *ZWF1* encoding glucose-6-phosphate dehydrogenase (G6PDH) was overexpressed in *S. cerevisiae*, the yeast strain could tolerate FF at a higher concentration than the respective wild type [53]. Direct connections between these genes in PPP pathway and FF tolerance have not been reported, yet it was speculated to be due to the availability of NADPH as this cofactor is essential for oxidative stress protection enzymes [15, 52, 53]. In addition to the aforementioned genes, *ADH1* was also reported to be related to FF tolerance in *Candida tropicalis* as it was shown that the ability to tolerate FF in this yeast decreased when *ADH1* was knocked out [49]. It should also be noted that the ability to reduce FF toxicity by converting FF into a less toxic form–furfuryl alcohol–could also contribute to such tolerances [12, 15].

As we known that FF can induces random mutations in yeast genomes [15, 21] and causes genetic alterations [22, 23]. FF might induce the mutations that affect the expression of genes related to the ethanol tolerance expression such as the vacuolar H^+^-ATPase and the plasma membrane H^+^-ATPase genes, which involved in reducing cytosolic acidification in yeast cells [50, 54], the heat shock proteins (HSPs) genes and trehalose metabolic enzyme genes, which played an important role in protecting protein structure denaturation from high concentration of ethanol [55–57] and *PUT4* genes encoding a high-affinity proline transporter, which increased proline uptake due to proline was able to protective effect against ethanol stress by reducing the ROS levels and increasing the survival rate of yeast cells [58, 59]. Altogether, this suggests that the casual mutations of AF2.5 by evolutionary engineering could be on one or more of these genes. However, to validate these assumptions, identification of the casual mutations should be investigated [60].

Likewise, several sets of genes are linked to ethanol tolerance including the ones responsible for cell envelope integrity and DNA damage repair [61]. Recently, transcriptome profiling indicating changes in gene regulations is reported upon ethanol stress and resulted in discontinuous metabolism and fermentation in *S. passalidarum* [62]. Thus, the observations made in this work could set a direction for future research, especially from evolutionary engineering point of view in *S. passalidarum*.

The adapted strain, AF2.5, entered the log phase faster than CMUWF1−2 in the presence of 2.0 and 3.0 g/l FF. The short lag phase in the presence of 2.0 g/l FF allowed AF2.5 to produce ethanol at high productivity (0.145±0.003 g/l/h), which is 1.48 times higher than from the wild type (data not shown). However, the highest ethanol titers in YPXyl were hardly different (Table 2). This could be because FF only affected growth-relating characteristics [18], yet these changes benefit ethanol production.

Long lag phases and reactive oxygen species (ROS) accumulation in yeast cells were induced by FF [18–20]. We showed that FF could induce ROS production in yeast cells by staining with H_2_DCFDA and measuring by a fluorescent microplate reader [41, 63]. Remarkably, AF2.5 accumulated the ROS in cells less than CMUWF1−2. This might be associated with changes in genes involved in the cell’s response to oxidative stress, such as those encoding superoxide dismutase or peroxiredoxin [64]. Furthermore, it might be involved in the expression of the superoxide detoxification gene (*SOD1*) upon ethanol stress, leading to a reduction in ROS, as reported in *S. passalidarum* [5].

It has been reported that the accumulation of intracellular ROS causes DNA damage [65]. The results showed that at 0 h, a high number of cells with diffuse nuclear chromatin were observed in both CMUWF1–2 and AF2.5 (around 43.32–54.45%). This may be attributed to the yeast cells in this study being freshly transferred to the test media and being in the phase of multiplication and adaptation, where transcription and translation are essential [47]. When the incubation time increased, the number of cells with tightly compacted chromatins increased in the absence of FF for both strains confirming the hypothesis mentioned; however, this was not the case for treatments with FF where the number of cells with compacted chromatins decreased suggesting the impact of FF on nuclear chromatin morphology. This is in agreement with Allen et al. [19], who reported that as FF concentration and incubation time increased, the nuclear chromatin in *S. cerevisiae* became more diffuse. This phenomenon was reported to be a result of the accumulation of ROS caused by FF, which, in turn, led to diffuse chromatin [19, 20]. Taken together, we hypothesize that the ability of AF2.5 to tolerate more FF and produce ethanol at higher productivity may be results from one or combined alterations mentioned above.

## Conclusions

In this study, the ALE approach was employed to significantly enhance furfural tolerance in *S. passalidarum* CMUWF1−2 by subjecting it to furfural as a single stressor. The duration of the ALE approach, which successfully yielded the adapted strain AF2.5, was shorter—consisting of only 17 rounds of transfer—compared to other research endeavors that typically utilized hydrolysate. Moreover, using furfural as a sole stressor resulted in the development of additional beneficial characteristics, including improved tolerances to ethanol (EtOH) and hydroxymethylfurfural (HMF), without compromising its inherent traits of good thermotolerance and absence of glucose repression. These findings provide valuable information into the development of ALE strategies for enhancing *S. passalidarum*, unlocking its potential to improve furfural tolerance and ethanol productivity for industrial applications.

### Electronic supplementary material

Below is the link to the electronic supplementary material.

Supplementary Material 1

## Data Availability

All data generated or analyzed during this study are included in this published article.
