# Supplementary material for: Improving furfural tolerance in a xylose-fermenting yeast Spathaspora passalidarum CMUWF1–2 via adaptive laboratory evolution
Source: Microb Cell Fact. 2024 Mar 13;23:80. doi: 10.1186/s12934-024-02352-x (PMC10936021; doi:10.1186/s12934-024-02352-x)
Supplement: Supplementary file 1 — Supplementary Material 1 [file 12934_2024_2352_MOESM1_ESM.docx]

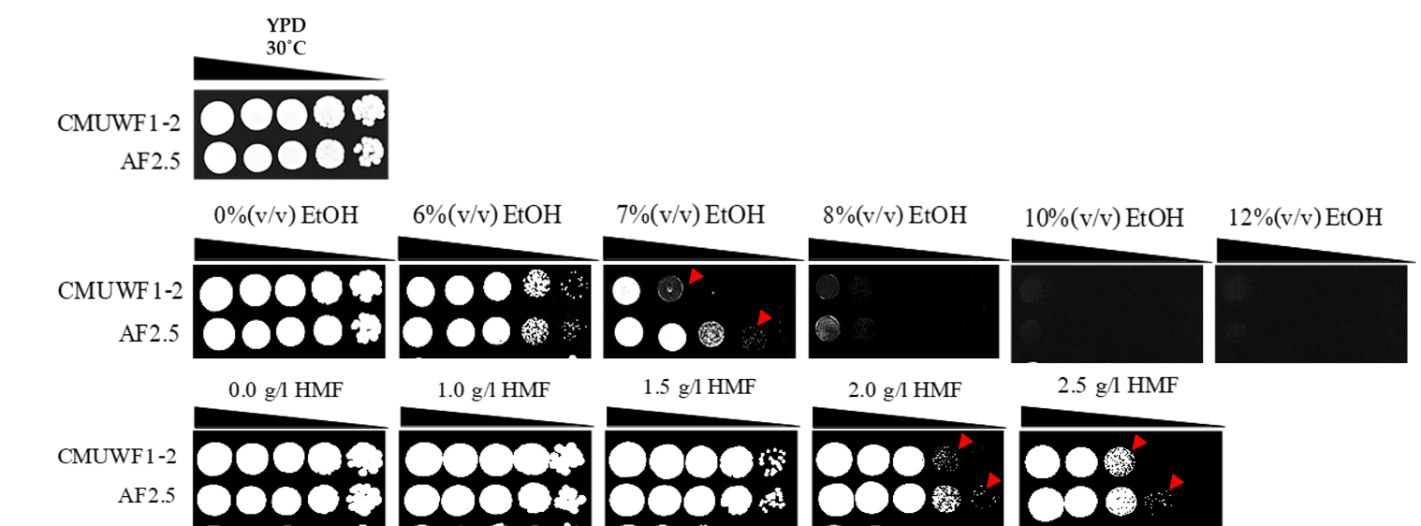


**Additional file 1: Fig. S1**

**Fig. S1** Comparison of stress tolerances of high concentrations of EtOH and HMF between *S. passalidarum* CMUWF1−2 and AF2.5. The cell suspension, containing approximately l × 10^7^ cells/ml was 10-fold serially diluted and spotted onto agar plates under different stresses. All plates were incubated at 30°C for 72 h. Data were reproduced by two independent experiments. Arrows indicate the presence of spotted cells.

**Additional file 2: Fig. S2**


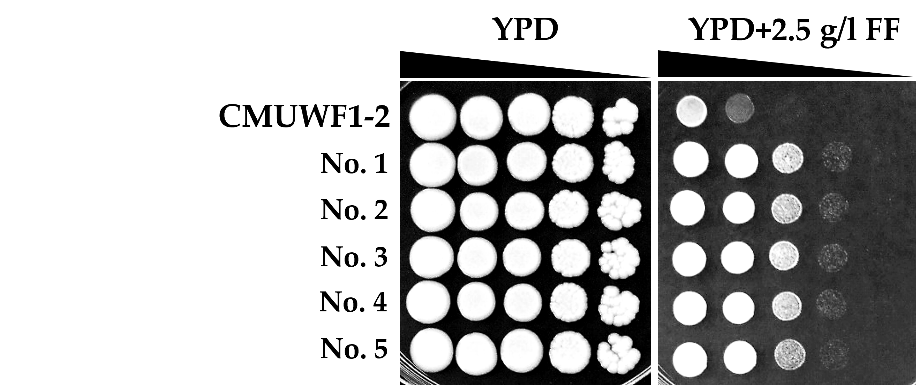


**Fig. S2** Comparison of FF tolerances between S. passalidarum CMUWF1−2 and adapted strains No.1-5. All strains were grown on YPD supplemented with 2.5 g/l FF at 30°C for 48 h. The cell suspension, containing approximately l × 10^7^ cells/ml was 10-fold serially diluted and spotted onto agar plates. Data were reproduced by two independent experiments.
